# Clinical assessment of VSR site and size and its relation to the severity of heart failure in post‐myocardial infarction ventricular septal rupture patients

**DOI:** 10.1002/clc.24062

**Published:** 2023-06-20

**Authors:** Ali Mansour Ali Nobah, Ezaldin M. I. Abuheit, Liguo Jian, Xiaofang Wang, Yanzhou Zhang

**Affiliations:** ^1^ Department of Cardiology First Affiliated Hospital of Zhengzhou University Zhengzhou Henan China; ^2^ Department of Cardiology Second Affiliated Hospital of Zhengzhou University Zhengzhou Henan China

**Keywords:** acute myocardial infarction, heart failure, post‐myocardial infarction, prodromal angina, ventricular septal rupture

## Abstract

**Background:**

Ventricular septal rupture (VSR) is a rare but well‐known mechanical consequence of an acute myocardial infarction (AMI). Even in the later stages of re‐perfusion therapy, the result of VSR remains poor. Our aim is to assess the site and size of VSR in relation to the severity of cardiac failure.

**Methods:**

From January 2016 to December 2022, a total of 71 patients with a diagnosis of post‐myocardial infarction VSR were admitted to the First Affiliated Hospital of Zhengzhou University, Zhengzhou, China. Data records were retrospectively included in this registry. In all patients, clinical and echocardiographic data were gathered, and statistical analyses were performed.

**Results:**

A total of 71 consecutive patients (mean age: 66.27 ± 8.88 years); 50.7% male, 49.3% female, with (M:F) ratio of almost (1:1). Left ventricular ejection fraction (LVEF) was (48.55 ± 10.44%) on echocardiography, and apical VSR was the most common site (69.0%). Overall, the VSD site was strongly related to the VSD size (*p* = .016), LVEF (*p* = .012), AMI site (*p* = .001), and affected coronary vessel (*p* = .004). Prodromal angina (*p* = .041), intra‐aortic balloon pump (*p* = .002), affected coronary vessels (*p* = .020), pro‐BNP (*p* = .000), and LVEF (*p* = .017) were predictors of the severity of heart failure.

**Conclusions:**

Diabetes mellitus is a common risk factor for post‐myocardial infarction VSR. VSR site and size had no relation to the severity of heart failure. A presentation with prodromal angina predicted severe heart failure and a worse prognosis.

## Graphical abstract

BACKGROUND: VSR is an uncommon but well‐recognized mechanical complication of AMI. The outcome of VSR remains poor even in the era of re‐perfusion therapy. PURPOSE: Our aim is to assess the site and size of ventricular septal rupture in relation to the severity of cardiac failure. METHODS: 71 patients with a diagnosis of post‐myocardial infarction ventricular septal rupture were retrospectively included in this registry. RESULTS: On echocardiography, the left ventricular ejection fraction was 48.55%, and the most frequent location of VSR was apical. Overall, the VSD site was strongly related to the VSD size. Prodromal angina, IABP, affected coronary vessels, pro‐BNP, and LVEF were predictors of the severity of heart failure. CONCLUSIONS: Diabetes mellitus is a common risk factor for post‐myocardial infarction ventricular septal rupture. VSR site and size had no relation to the severity of heart failure. A presentation with prodromal angina predicted severe heart failure and a worse prognosis.

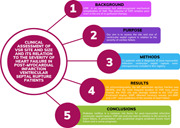

## 1 INTRODUCTION

A ventricular septal rupture (VSR) is an extremely serious and potentially fatal consequence of an acute myocardial infarction (AMI).^1^, ^2^ The incidence of VSR has drastically decreased since the introduction of effective reperfusion treatments (both pharmacological and mechanical). However, the fatality rate among patients with AMI and VSR is noted to be as high as 80% in a new sequence of cases with AMI undergoing immediate percutaneous coronary intervention (PCI), and appears relatively unaffected over the last few decades.^3^ The incidence of VSR following AMI was as high as 1–3 percent before the advent of thrombolysis and PCI.^4^, ^5^, ^6^, ^7^, ^8^ The incidence of VSR dropped to 0.17%–0.31%^9^, ^10^ after reperfusion therapies became the gold standard for treating AMI. The mortality rate from VSR remains exceedingly high, ranging from 45% to 80%,^11^, ^12^ despite advances in fast identification and treatment of both AMI and VSR.

As demonstrated by the SHOCK trial and confirmed by the GUSTO‐I and APEX‐AMI studies, VSR typically occurs much earlier, between 8 and 24 h after AMI, and the results did not differ substantially between those who received thrombolysis and those who did not.^9^, ^10^, ^11^, ^13^ However, the early detection of VSR may be the result of other factors, such as the widespread availability of echocardiography and changes in tissue pathology due to reperfusion injury and fibrinolysis.^3^, ^9^, ^14^ Despite improvements and optimistic results in nonsurgical treatments for VSR, such as transcatheter closure techniques,^15^ surgical repair of septal defects remains the primary treatment modality. The American College of Cardiology Foundation and American Heart Association's (ACCF/AHA) current guidelines suggest urgent surgical repair despite hemodynamic stability at the time of diagnosis.^16^

Despite professional understanding of the necessity of surgical repair, the timing of VSR repair and perioperative therapeutic management remain controversial.^4^, ^5^, ^7^, ^17^, ^18^ This research will evaluate the VSR site and size with particular regard to their relationship to the severity of heart failure.

## 2 METHODS

### 2.1 Medical ethics

This is a retrospective study involving data of human participants which is recruited from electronic medical records was reviewed and approved by The First Affiliated Hospital of Zhengzhou University and ethical number was not required.

### 2.2 Study design and patient selection

Seventy‐one patients who presented or were transferred to the First Affiliated Hospital of Zhengzhou University with VSR following AMI between January 2016 and December 2022 were analyzed retrospectively. Participants signed the patient permission form after a thorough description of the projects' aims, advantages, and even potential risks.

Any patient admitted for AMI who exhibited evidence of VSR on urgent cardiac catheterization or had hemodynamic compromise on echocardiography was considered for inclusion. Patients who did not survive their first hospital visit were not included; this included those who were rushed in for emergency cardiac catheterization but were unable to undergo it. Clinical symptoms and an elevation of serum troponin‐T >0.1 mg/dL were used to diagnose AMI, and an electrocardiographic (ECG) showing >2 mm ST‐segment elevation in the precordial leads or >1 mm ST‐segment elevation in the limb leads was also considered diagnostic. We reviewed all of the patients' medical records and analyzed their clinical profiles, therapies (both nonsurgical and surgical), and final results. Each patient's clinical hemodynamics were derived from the earliest available vital signs.

### 2.3 Cardiac catheterization

All patients who were diagnosed with AMI in the ER underwent immediate cardiac catheterization. The major goal of the coronary angiograms given to all patients was to initiate a primary intervention. Some patients also had a left ventriculogram before echocardiographic confirmation. The level of obstruction was used to describe coronary artery disease (CAD). No narrowing of more than 20% showed that there was no CAD. Nonobstructive coronary artery disease was diagnosed when there was at least one lesion with a narrowing of more than 20% but less than 70%. Obstructive CAD was described as stenosis of more than 70% or stenosis of the left main artery of more than 50% that affected one, two, or three vessels.

### 2.4 Echocardiography

All of the patients had an echocardiogram, and VSR was confirmed by transthoracic and/or transesophageal techniques within an average of 4 h and 36 min (up to 13 h and 51 min) of being admitted to the hospital. Color Doppler showed that there was a left‐to‐right backflow, which meant that the ventricular septum was broken. A transthoracic echocardiogram was used to find where the VSR was located. The VSR was found in the apical septum and the middle of the septum. Either the Quinonez method or Simpson's method with two planes were used to figure out the left ventricular ejection fraction (LVEF).

### 2.5 Variables

By reviewing the patient's medical records, we were able to learn about their demographic traits, medical co‐morbidities (such as smoking history, hypertension, diabetes mellitus, and renal function), medical acuity (such as prodromal angina, LVEF, and Killip and NYHA classifications), the location of the infarction, the site and size of the septal rupture, and the peak cardiac enzymes. Prodromal angina is defined as typical chest pain bouts lasting 30 min or longer that occur within 24 h of the AMI's onset (either at rest or with exertion).^19^ As additional variables for analysis, the intra‐aortic balloon pump (IABP), early PCI (6 h after AMI), concurrent surgical operations, and calendar year of operation were also taken into consideration.

### 2.6 Outcomes

The primary outcome was clinical correlation between the apex and middle VSR, defined as any factor that can differentiate the apical from the middle VSR based on clinical and statistical points. The secondary outcome was the indicators of severity of heart failure, defined as factors that can predict the severity of heart failure based on the clinical classification of cardiac failure and statistical analysis. The New York Heart Association classification of heart failure (Table S1) can be used to describe the severity of heart failure and the symptoms of heart failure.

### 2.7 Statistical analysis

Continuous variables were summarized as mean plus or minus the standard deviation (*SD*). Categorical variables were expressed as percentages of the sample. The comparison between apical VSR and middle VSR was performed by an independent sample test (Whitney *U* test) and the Chi‐square test using Yates correction and Fisher's exact test for continuous and categorical variables, respectively. A two‐tailed *p* < 0.05 was used to indicate statistical significance.

## 3 RESULTS

A total of 71 subjects were diagnosed with VSR after MI between the years of 2016–2022 (Table 1). The mean age ± *SD* was (66.2 ± 8.88) years old with (*n* = 36, 50.7%) were males and (*n* = 35, 49.3%) where females gave rise to (M:F) ratio almost equal (1.02:1). A total of 40 patients (56.3%) had diabetes mellitus, which was a common risk factor for post‐myocardial infarction VSR, followed by hypertension, which was found in (*n* = 33, 46.5%). A total of 26 patients (36.6%) were on dialysis. Smoking and a history of cerebrovascular accident were presented in 14 patients (19.7%) and 7 patients (9.9%), respectively. HDL was low in 21 patients (29.6%), and LDL was high in 17 patients (23.9%) (Table 1).

**Table 1:** Demographics and risk factors of VSR.

Variable	*n*	Value
Age, years (mean ± *SD*)	71	66.27 ± 8.88
Gender:	71	
Male	36	50.7
Female	35	49.3
Comorbidities:		
Diabetes mellitus	40	56.3
Hypertension	33	46.5
Dialysis	26	36.6
Smoking	14	19.7
History of CVA	7	9.9
Dyslipidemia:		
T.cho	5	7.0
TG	11	15.5
LDL	17	23.9
HDL	21	29.6

Abbreviations: CVA, cerebrovascular accident; HDL, high density lipoprotein; LDL, low density lipoprotein; SD, standard deviation; T.cho, total cholesterol; TG, triglycerides; VSR, ventricular septal rupture.

A total of 25 patients (35.2%) were supported by IABP (*n* = 19, 26.8%). The majority of patients at the time of diagnosis were Killip class III (*n* = 33, 46.5%) or IV (*n* = 20, 28.2%). Extensive anterior (*n* = 31, 43.7%) and anterior (*n* = 26, 36.6%) myocardial infarctions were the most common locations of myocardial infarction. The apical VSR (*n* = 49, 69.0%) was the most common VSR location, while there were 22 patients (31.0%) with the middle VSR. The average LVEF was (48.5 ± 10.4%).

A coronary angiogram was completed in all patients, with an unequal number of patients divided into 1‐, 2‐, or 3‐vessel disease categories. The left anterior descending coronary artery (LAD) was the most common location for culprit lesions (*n* = 60, 84.5%), followed by the right coronary artery (RCA) at *n* = 9, 12.7%). A revascularization procedure was performed in 65 patients. Among them, (*n* = 40, 56.3%) patients received PCI, and (*n* = 57, 80.3%) patients received thrombolysis (Table 2).

**Table 2:** Characteristics of 71 patients with **VSR** post **AMI**.

Variable	*n*	Value
Acuity:		
Mechanical support:		
IABP	25	35.2
ECMO	19	26.8
Prodromal angina	53	74.6
**Killip class**:		
**1**	7	9.9
**2**	11	15.5
**3**	33	46.5
**4**	20	28.2
**NYHA class**:		
**1**	–	–
**2**	3	4.2
**3**	22	31
**4**	46	64.8
Coronary artery disease:		
Single vessel disease	35	49.2
Double vessel disease	18	25.4
Multiple vessel disease	18	25.4
Culprit vessel lesion:		
LAD	60	84.5
LCX	2	2.8
RCA	9	12.7
**LVEF** (%) (mean ± *SD*)	71	48.55 ± 10.44
Cardiac enzymes:		
**CK**. (mean ± *SD*)	71	602.15 ± 911.29
**CK‐MB**. (mean ± *SD*)	71	57.61 ± 78.73
**Troponin T**. (mean ± *SD*)	71	2.04 ± 3.98
**Pro BNP**. (mean ± *SD*)	71	10926.49 ± 9900.60
**AMI site**:		
Anterior	26	36.6
Extensive anterior	31	43.7
Extensive anterolateral	5	7.0
Inferior	9	12.7
**VSR site**:		
Apex	49	69
Middle	22	31
**VSR size** (mean ± *SD*)	71	8.52 ± 4.56
Revascularization:		
PCI	40	56.3
Thrombolysis	57	80.3

Abbreviations: AMI, acute myocardial infarction; CK, creatinine kinase; CK‐MB, creatinine kinase MB fraction; ECMO, extra corporeal membrane oxygenation; IABP, intra‐aortic balloon pump; PCI, percutaneous coronary intervention; VSR, ventricular septal rupture.

**Figure 1:**
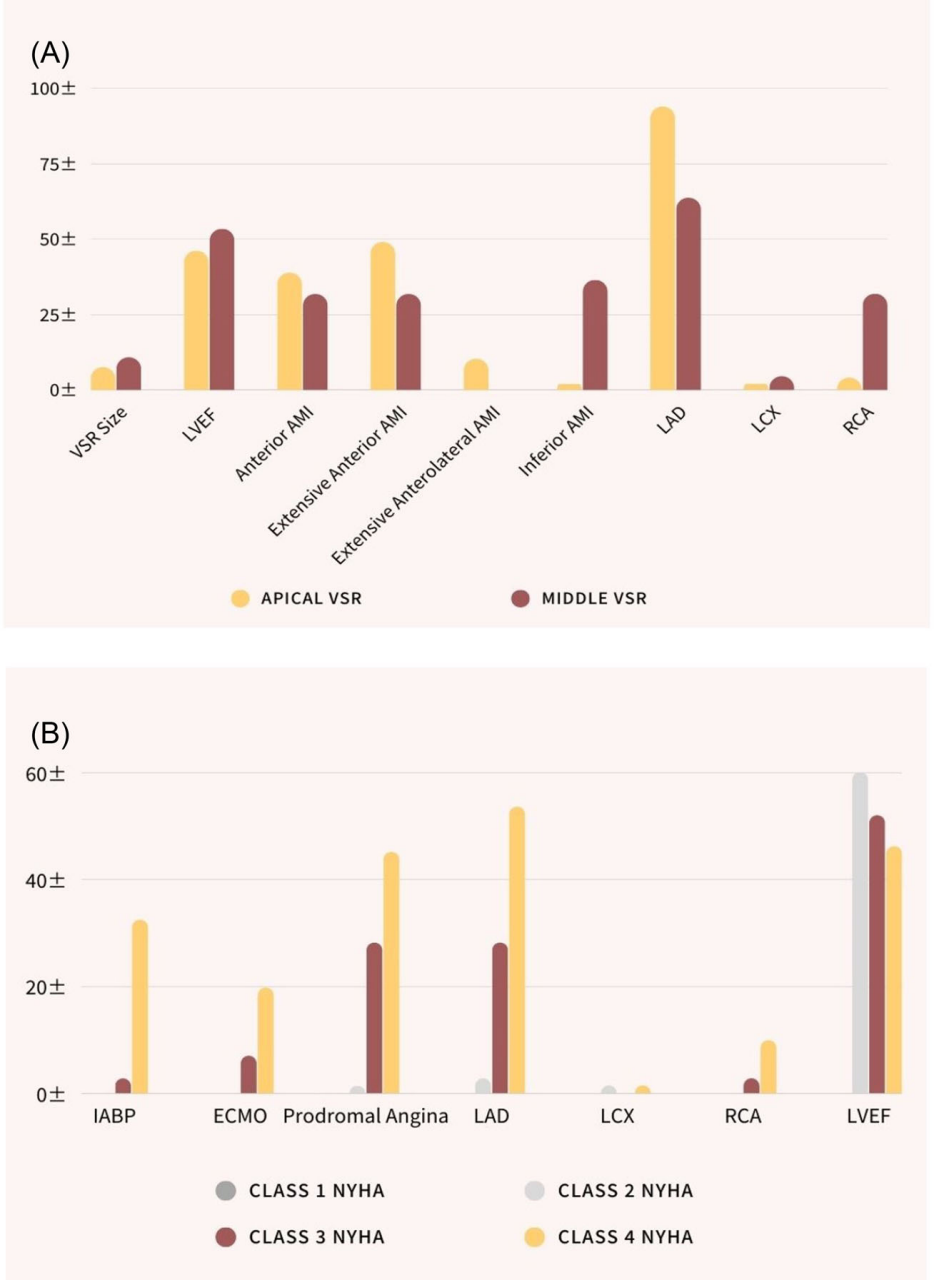
(A) Significant factors predicted the difference between apical and middle VSR. (B) Significant factors predicted the severity of heart failure. VSR, ventricular septal rupture.

From Table 3, age, gender, killip class, NYHA class, and Pro‐BNP were not significantly different between the apical VSR group and the middle VSR group. The apical VSR group had a lower LVEF than the middle VSR group (46.08 ± 8.88% vs. 53.36 ± 11.72%, *p* = .012); a lower LVEF predicted severe heart failure. There was a significant difference in the size of the VSR among the two groups, which was smaller in the apical group than that in the middle one (7.51 ± 3.37 mm vs. 10.77 ± 5.97 mm, *p* = .016). The location of the myocardial infarction had a significant difference among the two groups with a p value was (*p* = .001). This strong variance showed that the apical VSR almost always comes with the anterior wall myocardial infarction, while the middle VSR mostly comes with the inferior wall myocardial infarction. The affected coronary artery vessel was almost the left anterior descending coronary artery (LAD) in the apical group, while the left anterior descending coronary artery (LAD) and the RCA were the most common in the middle group with a significant difference was (*p* = .004) (Table 3) (Figure 1A).

**Table 3:** Comparison between apical and middle VSR.

Variable	Apical VSR (*n* = 49, 69%)		Middle VSR (*n* = 22, 31.0%)		*p* value
	*n*	Value	*n*	Value	
Age, years	49	66.69 ± 9.03	22	65.32 ± 8.67	.332
Gender:					.664
Male	24	48.98	12	54.54	
Female	25	51.02	10	45.46	
VSR size (mean ± *SD*)	49	7.51±3.37	22	10.77± 5.97	**.016**
LVEF (%) (mean ± *SD*)	49	46.08 ±8.98	22	53.36± 11.72	**.012**
Killip class:					
1	5	10.2	2	9.1	
2	5	10.2	6	27.3	
3	23	46.9	10	45.5	
4	16	32.7	4	18.2	.260
NYHA class:					
1	–	–	–	–	
2	2	4.1	1	4.5	
3	13	26.5	7	31.9	
4	34	69.4	14	63.6	.890
Pro‐BNP (mean ± *SD*)	49	11088.74±10127.73	22	10564.86± 9596.63	.960
AMI site:					
Anterior	19	38.8	7	31.8	
Extensive anterior	24	49.0	7	31.8	
Extensive anterolateral	5	10.2	–	–	
Inferior	1	2.0	8	36.4	**.001**
Affected vessel:					
LAD	46	93.9	14	63.7	
LCX	1	2.0	1	4.5	
RCA	2	4.1	7	31.8	**.004**

*Note*: Bold values indicate significant *p* value for the factors that showed clinical significance.

Abbreviations: LAD, left anterior descending coronary artery; LCX, circumflex artery; LVEF, left ventricular ejection fractions; NYHA, New York heart association; Pro‐BNP, N‐terminal portion pro brain natriuretic peptide; RCA, right coronary artery; VSR, ventricular septal rupture.

A total of 71 patients were distributed over classes II, III, and IV of the New York Heart Association classification of heart failure (NYHA). Here we describe the relationship between VSR and heart failure based on the clinical classification. There were no patients in class I; only 3 patients were in class II; 22 patients were in class III; and 46 patients were in class IV. As it is shown in Table 4, age, gender, VSR site, and size had no significant difference between each class of heart failure. Mechanical support with the IABP had a significant difference between classes III and IV (*n* = 2, 2.82% vs. *n* = 23, 32.4%, *p* = .002), while mechanical support with extra corporeal membrane oxygenation (ECMO) had a marginally significant difference (*p* = .056). Prodromal angina had also a significant difference between classes II, III, and IV with a p value was (*p* = .041; this strong difference predicted the severity of heart failure. There was a significant difference in the affected vessel among the 3 classes of heart failure (*p* = .020). Pro‐BNP had also a strong variance between the 3 classes of heart failure, with a *p* value was (*p* = .000), which indicated that the higher the Pro‐BNP value, the more severe the heart failure. And last, the LVEF was significantly different among the 3 classes of heart failure (*p* = .017), which predicted that the lower the LVEF, the higher the stage of heart failure (Table 4) (Figure 1B).

**Table 4:** The relationship of ventricular septal rupture to the severity of the heart failure.

**Variable**	Class 1 NYHA	Class 2 NYHA		Class 3 NYHA		Class 4 NYHA		*p* value
	(*n* = 0, value)	(*n*=3, value)		(*n* = 22, value)		(*n* = 46, value)		
Age, years	–	71.0 ± 6.0		66.27 ± 9.84		65.96 ± 8.61		.583
Gender:								.778
Male	–	1	1.4	12	16.8	23	32.4	
Female	–	2	2.82	10	14.1	23	32.4	
Mechanical support:								
IABP		0	0	2	2.82	23	32.4	**.002**
ECMO	–	0	0	5	7.04	14	19.72	.056
Prodromal angina	–	1	1.4	20	28.17	32	45.07	**.041**
Affected vessel:								**.020**
LAD	–	2	2.82	20	28.17	38	53.52	
LCX		1	1.4	–	–	1	1.4	
RCA	–	–	–	2	2.82	7	9.86	
VSR site:	–							.989
Apex	–	2	2.82	15	21.13	32	45.07	
Middle	–	1	1.4	7	9.86	14	19.72	
VSR size		6.67 ± 2.30		7.45 ± 2.84		9.11 ± 5.25		.535
Pro‐BNP	–	3977.3 ± 696.67		4883.7 ± 4005.7		14269.57 ± 10605.4		**.000**
LVEF	–	60.0 ± 5.29		51.95 ± 10.98		46.17 ± 9.58		**.017**

*Note*: Definition: prodromal Angina: was defined as typical chest pain episodes (either at rest or upon effort) persisting <30 min and occurring within 24 h before the onset of the AMI.^19^ Bold values indicate significant *p* value for the factors that showed clinical significance.

Abbreviations: AMI, acute myocardial infarction; ECMO, extra corporeal membrane oxygenation; LAD, left anterior descending coronary artery; LCX, circumflex artery; LVEF, left ventricular ejection fractions; NYHA, New York heart association; Pro‐BNP, N‐terminal portion pro brain natriuretic peptide; RCA, right coronary artery; VSR, ventricular septal rupture.

## 4 DISCUSSION

While reading the research conclusions, individual points are worth pointing out. First, modern reperfusion strategies and more potent antiplatelet therapies have modified the epidemiology of VSR complicating AMI.^3^ In fact, the frequency of this complication has greatly decreased and is accounted for as high as 0.2% in recent primary PCI studies.^20^ In the current series, we found a slightly higher incidence of VSR complicating AMI (0.77%). This registry includes participants admitted to a tertiary medical center where patients have high disease severity. In addition, some victims with AMI were passed on to our center due to the diagnosis of VSR, a phenomenon that could have inflated the recognized incidence of this mechanical complication.

Second, necropsy studies of patients experiencing fatal AMI have frequently demonstrated that VSR occurs as a consequence of a transmural infarction.^21^ In this consideration, the reduction of ischemia time among patients with AMI pursued in the last few decades in Western states has generally reduced the prevalence and cut down the time to diagnosis of VSR complicating AMI.^20^ Indeed, recent data confirm that VSR complicating AMI is diagnosed earlier (within 24 h) as compared with historical cohorts (3–5 days).^3^ In the current report, 27.4% of the patients received reperfusion therapy, and the median time interval between AMI and VSR diagnosis was 3 days. There were no baseline differences between patients undergoing reperfusion and those who did not. The efforts in reducing delays, facilitating access to hospitals, and providing affordable treatments for patients with AMI should represent the main objectives of care professionals to lower the burden of morbidity and mortality associated with cardiovascular disease.

Age, gender, Killip class, NYHA class, and pro‐BNP had no significant variance between the two groups (apical and middle VSR), which indicated no correlation between these factors and the site of the VSR. There was a significant difference in the size of the VSR among the two groups, which was smaller in the apical group than that in the middle one. LVEF can be used as an indicator to differentiate between apical and middle VSR, as it was lower in the apical VSR than that in the middle one, unless the size of the middle VSR was >10 mm, which is common as it is mentioned above, with a significant p value. The site of the AMI and the affected coronary artery were also significant. The apical and middle VSR can be roughly clinically differentiated from each other by the site of the AMI that appears on the ECG paper, the apical VSR most commonly coming with the extensive anterior AMI followed by anterior AMI, while the middle VSR was mostly coming with the inferior myocardial infarction followed by the anterior one; the left anterior descending (LAD) coronary artery was mostly coming with the apical VSR while the RCA was not uncommon with the middle VSR.

This study confirmed that age, gender, site, and size of the VSR had no significance, which indicated that these factors were not affecting the severity of heart failure. Mechanical support with the IABP and ECMO were significant and marginally significant, respectively. Case reports and other small retrospective studies demonstrate that the use of IABP improves survival following VSR until surgical repair.^22^, ^23^ In a large retrospective investigation of 2876 cases from the Society of Thoracic Surgeons Adult Cardiac Surgery Database conducted by Arnaoutakis et al.,^24^ the use of IABP both preoperatively and intraoperatively was associated with substantially higher mortality rates. The data on the use of ECMO in VSR have also been limited to case studies, and there have been no prospective or retrospective studies evaluating the mortality benefits of VSR after MI.^25^, ^26^ ACC/AHA guidelines^3^ recommend the use of IABP in patients with cardiogenic shock due to VSR.

Our findings suggest that both IABP and EMCO provide survival advantages through preoperative hemodynamic support and postoperative advantages with the use of EMCO. Individuals who had prodromal angina frequently have a stage III or IV NYHA classification, so prodromal angina revealed significant variation and anticipated the severity of heart failure. The left anterior descending (LAD) coronary artery almost always takes place with stage IV heart failure, followed by the RCA, as proved in previous studies. Pro‐BNP had also a great variance between the 3 classes of heart failure, which indicated that the greater the Pro‐BNP value, the more severe the heart failure. And last, the LVEF was significantly different among the 3 classes of heart failure, which predicted that the lower the LVEF, the higher the stage of heart failure.

## 5 CONCLUSIONS

1. There is no relationship between the site and size of the VSR and the severity of heart failure.
2. Presentation with prodromal angina indicates more severe heart failure and a worse prognosis.
3. Certain factors (VSR size, LVEF, AMI site, and affected coronary artery) can give a clue to the site of the VSR.
4. Mechanical support with IABP and ECMO, prodromal angina, affected coronary arteries, pro‐BNP, and LVEF are directly proportionate to the severity of heart failure.
5. Diabetes mellitus is a common risk factor for post‐myocardial infarction VSR.

## AUTHOR CONTRIBUTIONS

All authors listed have made a substantial, direct and intellectual contribution to the work, and approved it for publication.

## CONFLICT OF INTEREST STATEMENT

The authors declare no conflicts of interest.

## Supporting information

Supporting information.

## Data Availability

The data that support the findings of this study are available on request from the corresponding author. The data are not publicly available due to privacy or ethical restrictions.
