# Supplementary material for: Clinical assessment of VSR site and size and its relation to the severity of heart failure in post‐myocardial infarction ventricular septal rupture patients
Source: Clin Cardiol. 2023 Jun 20;46(8):981–8. doi: 10.1002/clc.24062 (PMC10436785; doi:10.1002/clc.24062)
Supplement: Supplementary file 1 — Supporting information. [file CLC-46-981-s001.docx]

**Supplementary table 1:** New York Heart Association (NYHA) Classification of Heart Failure.

| Class I | No limitation. Normal physical exercise does not cause fatigue, dyspnea or palpitations |
| --- | --- |
| Class II | Mild limitation. Comfortable at rest but normal physical activity produces fatigue, dyspnea or palpitations |
| Class III | Marked limitation. Comfortable at rest but gentle physical activity produces marked symptoms of heart failure |
| Class IV | Symptoms of heart failure occur at rest and are exacerbated by any physical activity |
